# Supplementary material for: MetaRibo-Seq measures translation in microbiomes
Source: Nat Commun. 2020 Jun 29;11:3268. doi: 10.1038/s41467-020-17081-z (PMC7324362; doi:10.1038/s41467-020-17081-z)
Supplement: Supplementary file 10 — Supplementary Data 7 [file 41467_2020_17081_MOESM10_ESM.zip › File2/Confidence_High_Taxonomy/319807_out.krona.html]

Javascript must be enabled to view this page.

members
magnitude
magnitudeUnassigned
count
unassigned
taxon
rank

319807\_out

343

superkingdom
2
343

343
976
phylum

class
200643
343

order
171549
343

343
815
family

343
33
816

SRS014459\_contig\_number\_contig-100\_3732.221711SRS015694\_contig\_number\_23211SRS016267\_contig\_number\_12945SRS018351\_contig\_number\_14316SRS018836\_contig\_number\_2352SRS018936\_contig\_number\_contig-100\_3516.91406SRS019161\_contig\_number\_contig-100\_1781.151569SRS020271\_contig\_number\_3797SRS021219\_contig\_number\_13271SRS022137\_contig\_number\_contig-100\_3126.113446SRS024435\_contig\_number\_contig-100\_34008.115992SRS024625\_contig\_number\_21392SRS045713\_contig\_number\_22323SRS047014\_contig\_number\_8092SRS047044\_contig\_number\_1342SRS050520\_contig\_number\_contig-100\_10411.10412SRS055017\_contig\_number\_1983SRS075878\_contig\_number\_2173SRS077730\_contig\_number\_18120SRS078242\_contig\_number\_contig-100\_2770.180718SRS103987\_contig\_number\_5382SRS1041112\_contig\_number\_5801SRS1041132\_contig\_number\_974SRS1055067\_contig\_number\_9477SRS147022\_contig\_number\_contig-100\_2192.2192SRS147022\_contig\_number\_contig-100\_10480.72479SRS147139\_contig\_number\_contig-100\_15182.187348SRS147727\_contig\_number\_contig-100\_8197.8198SRS148091\_contig\_number\_3497SRS148970\_contig\_number\_4315SRS893279\_contig\_number\_17609SRS893292\_contig\_number\_344SRS893358\_contig\_number\_contig-100\_4589.4590
genus


SRS075021\_contig\_number\_6878
species
2292193
1


SRS013476\_contig\_number\_12674SRS014736\_contig\_number\_9259SRS016056\_contig\_number\_7783SRS018427\_contig\_number\_32269SRS018575\_contig\_number\_5051SRS020508\_contig\_number\_1038SRS022713\_contig\_number\_1556SRS023583\_contig\_number\_17294SRS045826\_contig\_number\_251SRS046810\_contig\_number\_contig-100\_77.78SRS048870\_contig\_number\_4829SRS050801\_contig\_number\_contig-100\_311.35773SRS052576\_contig\_number\_127SRS054905\_contig\_number\_4134SRS054956\_contig\_number\_1644SRS056273\_contig\_number\_3756SRS056519\_contig\_number\_1921SRS057478\_contig\_number\_5311SRS063040\_contig\_number\_760SRS063489\_contig\_number\_6694SRS063518\_contig\_number\_8099SRS064557\_contig\_number\_5437SRS065397\_contig\_number\_8644SRS075078\_contig\_number\_31170SRS076756\_contig\_number\_19116SRS078419\_contig\_number\_28607SRS098717\_contig\_number\_16239SRS101376\_contig\_number\_27316SRS1041136\_contig\_number\_25534SRS1041137\_contig\_number\_contig-100\_470.170165SRS1041138\_contig\_number\_3945SRS1041164\_contig\_number\_5822SRS104485\_contig\_number\_1168SRS142923\_contig\_number\_14376SRS143523\_contig\_number\_21084SRS143991\_contig\_number\_27703SRS146813\_contig\_number\_9051SRS147919\_contig\_number\_2050SRS147977\_contig\_number\_9052SRS148424\_contig\_number\_35827SRS892882\_contig\_number\_16783SRS893259\_contig\_number\_6799SRS893373\_contig\_number\_26323
species
1739517
43

127

SRS012273\_contig\_number\_38041SRS012849\_contig\_number\_33084SRS012902\_contig\_number\_726SRS013098\_contig\_number\_33112SRS013215\_contig\_number\_949SRS013638\_contig\_number\_16917SRS013940\_contig\_number\_10087SRS014683\_contig\_number\_3357SRS014855\_contig\_number\_15048SRS015264\_contig\_number\_2947SRS015369\_contig\_number\_6756SRS015579\_contig\_number\_1992SRS015782\_contig\_number\_15456SRS015794\_contig\_number\_12242SRS015854\_contig\_number\_24148SRS015890\_contig\_number\_13482SRS015960\_contig\_number\_4617SRS016018\_contig\_number\_10539SRS016132\_contig\_number\_375SRS016495\_contig\_number\_15663SRS016840\_contig\_number\_2787SRS017191\_contig\_number\_10861SRS017433\_contig\_number\_9843SRS017701\_contig\_number\_19587SRS017859\_contig\_number\_7008SRS018313\_contig\_number\_7432SRS018623\_contig\_number\_1164SRS018817\_contig\_number\_11767SRS018984\_contig\_number\_6099SRS019030\_contig\_number\_11260SRS019178\_contig\_number\_5651SRS019601\_contig\_number\_11629SRS019693\_contig\_number\_667SRS020328\_contig\_number\_22908SRS020394\_contig\_number\_17223SRS022071\_contig\_number\_29386SRS022524\_contig\_number\_15248SRS023346\_contig\_number\_4218SRS023526\_contig\_number\_20705SRS023715\_contig\_number\_27874SRS023829\_contig\_number\_673SRS023914\_contig\_number\_28899SRS023971\_contig\_number\_779SRS024075\_contig\_number\_14861SRS024388\_contig\_number\_8509SRS024549\_contig\_number\_4466SRS024663\_contig\_number\_13980SRS042628\_contig\_number\_3495SRS043001\_contig\_number\_9643SRS043701\_contig\_number\_2197SRS043841\_contig\_number\_11442SRS045004\_contig\_number\_19400SRS045645\_contig\_number\_29850SRS046712\_contig\_number\_2491SRS046717\_contig\_number\_2806SRS048164\_contig\_number\_12683SRS050026\_contig\_number\_5904SRS050422\_contig\_number\_36700SRS050941\_contig\_number\_19619SRS051882\_contig\_number\_21031SRS053214\_contig\_number\_16756SRS053649\_contig\_number\_7979SRS054352\_contig\_number\_contig-100\_202.138986SRS056537\_contig\_number\_46749SRS057717\_contig\_number\_499SRS058070\_contig\_number\_4395SRS058268\_contig\_number\_105SRS062427\_contig\_number\_1480SRS064232\_contig\_number\_1463SRS065176\_contig\_number\_16966SRS065504\_contig\_number\_38161SRS074964\_contig\_number\_1488SRS075398\_contig\_number\_903SRS075773\_contig\_number\_22820SRS075821\_contig\_number\_18205SRS075984\_contig\_number\_25871SRS076804\_contig\_number\_2359SRS077231\_contig\_number\_26073SRS077454\_contig\_number\_14025SRS077502\_contig\_number\_14614SRS078665\_contig\_number\_1155SRS097958\_contig\_number\_21606SRS098571\_contig\_number\_44421SRS098655\_contig\_number\_13054SRS098881\_contig\_number\_15584SRS104084\_contig\_number\_1072SRS1041032\_contig\_number\_6404SRS1041037\_contig\_number\_12947SRS1041095\_contig\_number\_5941SRS1041134\_contig\_number\_9180SRS1041139\_contig\_number\_6698SRS104311\_contig\_number\_19050SRS104327\_contig\_number\_contig-100\_429.47137SRS1055034\_contig\_number\_contig-100\_1626.73720SRS1055043\_contig\_number\_14047SRS1055056\_contig\_number\_13896SRS1055069\_contig\_number\_6064SRS1055083\_contig\_number\_3228SRS1055095\_contig\_number\_970SRS140645\_contig\_number\_154SRS140645\_contig\_number\_2095SRS142542\_contig\_number\_1701SRS142980\_contig\_number\_5491SRS143466\_contig\_number\_26477SRS143722\_contig\_number\_1039SRS143780\_contig\_number\_13211SRS144297\_contig\_number\_14034SRS144506\_contig\_number\_51797SRS144714\_contig\_number\_contig-100\_5586.139963SRS145308\_contig\_number\_1369SRS145497\_contig\_number\_15691SRS147039\_contig\_number\_4350SRS147139\_contig\_number\_25783SRS147272\_contig\_number\_979SRS147653\_contig\_number\_2052SRS148196\_contig\_number\_37527SRS148253\_contig\_number\_12457SRS148817\_contig\_number\_13693SRS149244\_contig\_number\_5502SRS149784\_contig\_number\_23764SRS893170\_contig\_number\_8861SRS893231\_contig\_number\_6613SRS893285\_contig\_number\_1504SRS893327\_contig\_number\_582SRS893342\_contig\_number\_8986SRS893369\_contig\_number\_8873SRS971276\_contig\_number\_29202
species
28116

species

SRS015217\_contig\_number\_25775SRS1055099\_contig\_number\_14871
204516
2

species

SRS063190\_contig\_number\_13247SRS1055022\_contig\_number\_5352
469590
2

6

SRS014613\_contig\_number\_327SRS045739\_contig\_number\_10850SRS056259\_contig\_number\_contig-100\_173.35202SRS098073\_contig\_number\_1425SRS140513\_contig\_number\_2575SRS143148\_contig\_number\_5737
species
818

species

SRS016753\_contig\_number\_4662SRS049446\_contig\_number\_8881
291644
2

585544

SRS014415\_contig\_number\_contig-100\_3901.3902SRS016954\_contig\_number\_25623SRS019397\_contig\_number\_9325SRS019808\_contig\_number\_6286SRS044535\_contig\_number\_21722SRS097920\_contig\_number\_14861SRS104912\_contig\_number\_19487
species
7

46506

SRS1055076\_contig\_number\_14325
species
1

2292957

SRS014287\_contig\_number\_4200SRS015190\_contig\_number\_10606SRS015578\_contig\_number\_30789SRS045244\_contig\_number\_2600SRS055982\_contig\_number\_11992SRS063928\_contig\_number\_43SRS077552\_contig\_number\_22254SRS1041090\_contig\_number\_7968SRS1041144\_contig\_number\_2180SRS893300\_contig\_number\_11426SRS893366\_contig\_number\_20187SRS893383\_contig\_number\_35469
species
12

36
371601

SRS012969\_contig\_number\_39936SRS013382\_contig\_number\_contig-100\_48.27405SRS013800\_contig\_number\_10674SRS014534\_contig\_number\_2448SRS016897\_contig\_number\_1867SRS016989\_contig\_number\_9767SRS017307\_contig\_number\_16315SRS018049\_contig\_number\_630SRS018133\_contig\_number\_10151SRS018541\_contig\_number\_4698SRS019286\_contig\_number\_3728SRS020233\_contig\_number\_12282SRS020869\_contig\_number\_11759SRS021153\_contig\_number\_385SRS022609\_contig\_number\_51437SRS024009\_contig\_number\_7045SRS045195\_contig\_number\_367SRS047433\_contig\_number\_19285SRS050998\_contig\_number\_contig-100\_688.102377SRS051610\_contig\_number\_2882SRS075341\_contig\_number\_19433SRS075716\_contig\_number\_1846SRS077589\_contig\_number\_4919SRS101268\_contig\_number\_117SRS1041036\_contig\_number\_2020SRS1041133\_contig\_number\_20875SRS1041145\_contig\_number\_39811SRS1041146\_contig\_number\_61SRS104636\_contig\_number\_22374SRS1055049\_contig\_number\_13466SRS142599\_contig\_number\_21434SRS147271\_contig\_number\_16424SRS149325\_contig\_number\_14484SRS893295\_contig\_number\_12121SRS893341\_contig\_number\_11386SRS893380\_contig\_number\_1316
species


SRS142781\_contig\_number\_145
species
820
1

1

SRS097889\_contig\_number\_15478
species
2292951

2

SRS014412\_contig\_number\_5458SRS143598\_contig\_number\_4586
47678
species


SRS043667\_contig\_number\_5308
457390
species
1

species

SRS018888\_contig\_number\_5463SRS053335\_contig\_number\_15769SRS147766\_contig\_number\_503SRS149879\_contig\_number\_12310
457387
4


SRS017622\_contig\_number\_100
2292956
species
1

1
1262750

SRS1041130\_contig\_number\_1827
species


SRS049773\_contig\_number\_21394SRS077127\_contig\_number\_15193SRS893288\_contig\_number\_1068
1263050
species
3


SRS019787\_contig\_number\_4099SRS043411\_contig\_number\_293SRS053573\_contig\_number\_391SRS143417\_contig\_number\_17633SRS148159\_contig\_number\_43841
species
817
5

1

SRS020622\_contig\_number\_1867
species
674529

1

SRS143181\_contig\_number\_3194
species
310297


SRS1041116\_contig\_number\_6934
species
2302926
1

species

SRS056519\_contig\_number\_7368
376806
1

48
2292955

SRS011134\_contig\_number\_36317SRS011239\_contig\_number\_3753SRS011405\_contig\_number\_14965SRS013158\_contig\_number\_contig-100\_71.36288SRS013687\_contig\_number\_34716SRS013951\_contig\_number\_38498SRS014923\_contig\_number\_contig-100\_979.238367SRS016095\_contig\_number\_27360SRS016335\_contig\_number\_1632SRS016381\_contig\_number\_contig-100\_664.30269SRS017103\_contig\_number\_42178SRS024132\_contig\_number\_22309SRS024331\_contig\_number\_37119SRS046068\_contig\_number\_contig-100\_426.35172SRS046369\_contig\_number\_contig-100\_712.107693SRS048981\_contig\_number\_6712SRS049896\_contig\_number\_25931SRS050752\_contig\_number\_24311SRS050925\_contig\_number\_12346SRS052078\_contig\_number\_4376SRS057480\_contig\_number\_contig-100\_810.75653SRS058770\_contig\_number\_25605SRS062654\_contig\_number\_18197SRS062701\_contig\_number\_1525SRS063813\_contig\_number\_contig-100\_1556.54010SRS064276\_contig\_number\_39506SRS074670\_contig\_number\_1660SRS076929\_contig\_number\_contig-100\_1738.153741SRS077849\_contig\_number\_30907SRS098644\_contig\_number\_46418SRS1041031\_contig\_number\_contig-100\_1358.143303SRS1041039\_contig\_number\_4048SRS1041142\_contig\_number\_4559SRS104197\_contig\_number\_contig-100\_356.3406SRS1054928\_contig\_number\_contig-100\_1752.43588SRS1055038\_contig\_number\_3461SRS142503\_contig\_number\_contig-100\_2077.300275SRS142890\_contig\_number\_7739SRS143070\_contig\_number\_231SRS143085\_contig\_number\_5433SRS143876\_contig\_number\_41118SRS143895\_contig\_number\_contig-100\_2043.249304SRS144362\_contig\_number\_644SRS147022\_contig\_number\_4629SRS147614\_contig\_number\_2878SRS148784\_contig\_number\_26047SRS148874\_contig\_number\_14028SRS150029\_contig\_number\_17202
species
